# Extracerebral metastases determine the outcome of patients with brain metastases from renal cell carcinoma

**DOI:** 10.1186/1471-2407-10-480

**Published:** 2010-09-07

**Authors:** Ursula M Vogl, Marija Bojic, Wolfgang Lamm, Josa M Frischer, Oskar Pichelmayer, Gero Kramer, Andrea Haitel, Klaus Kitz, Kaan Harmankaya, Christoph C Zielinski, Manuela Schmidinger

**Affiliations:** 1 Department of Medicine I, Clinical Division of Oncology and Cancer Center, Medical University Vienna, Vienna, Austria; 2 Department of Neurosurgery, Medical University Vienna, Vienna, Austria; 3 Department of Urology, Medical University Vienna, Vienna, Austria; 4 Department of Pathology, Medical University Vienna, Vienna, Austria; 5 Department of Dermatology, Medical University Vienna, Vienna, Austria

## Abstract

**Background:**

In the era of cytokines, patients with brain metastases (BM) from renal cell carcinoma had a significantly shorter survival than patients without. Targeted agents (TA) have improved the outcome of patients with metastatic renal cell carcinoma (mRCC) however, their impact on patients with BM is less clear. The aim of this analysis was to compare the outcome of patients with and without BM in the era of targeted agents.

**Methods:**

Data from 114 consecutive patients who had access to targeted agent were analyzed for response rates (ORR), progression free survival (PFS) and overall survival (OS). All patients diagnosed with BM underwent local, BM-specific treatment before initiation of medical treatment.

**Results:**

Data of 114 consecutive patients who had access to at least one type of targeted agents were analyzed. Twelve out of 114 renal cell carcinoma (RCC) patients (10.5%) were diagnosed with BM. Systemic treatment consisted of sunitinib, sorafenib, temsirolimus or bevacizumab. The median PFS was 8.7 months (95% CI 5.1 - 12.3) and 11.4 months (95% CI 8.7 - 14.1) for BM-patients and non-BM-patients, respectively (p = 0.232). The median overall survival for patients with and without BM was 13.4 (95% CI 1- 43.9) and 33.3 months (95% CI 18.6 - 47.0) (p = 0.358), respectively. No patient died from cerebral disease progression. ECOG Performance status and the time from primary tumor to metastases (TDM) were independent risk factors for short survival (HR 2.74, p = 0.001; HR: 0.552, p = 0.034).

**Conclusions:**

Although extracerebral metastases determine the outcome of patients with BM, the benefit from targeted agents still appears to be limited when compared to patients without BM.

## Background

Brain metastases (BMs) are observed in 2% to 17% of patients with metastatic renal cell carcinoma (mRCC) [1]. The majority of these patients present with metastatic disease in multiple organs. Despite the availability of several local treatment strategies for BMs, such as conventional surgery, whole brain radiation therapy (WBRT) and stereotactic radiation (SR), the prognosis of these patients is poor. Patients with BMs were reported to have a median overall survival of 4-5 months after diagnosis and treatment of cerebral lesions [2].

Upon diagnosis of BM, patients usually undergo neurosurgical and/or radiotherapeutic procedures whereas medical treatment may be offered later. The choice for a specific type of local treatment depends on the size and number of BMs, their intracerebral location and the patient's condition. Historical data have shown that stereotactic radiation for BMs from renal cell carcinoma (RCC) may result in brain-specific disease control up to 10 months [3,4]. Another effective local treatment option is surgery. Most brain metastases from renal cell carcinoma (RCC) are well circumscribed and relatively firm, which makes them suitable for complete surgical resection. Surgical resection was shown to enable a median overall survival of more than one year [1,5]. In contrast, whole brain radiation appears to confer the smallest benefit in terms of median time to local disease progression (3 to 8 months) [5].

Although surgery or stereotactic radiation may be highly effective to reduce the risk of brain-specific disease progression, BM-patients were shown to have a shorter survival than patients without BM [6]. Interestingly, several authors observed that this was related to the lack of effective medical treatment options with rapid extracerebral disease progression rather than to BM-associated complications such as bleeding or increased brain pressure [1,2,5,7]. BM-patients were mostly considered unsuitable for cytokines and only few reports indicate that interferon-alpha (IFN-alpha) and/or interleukin-2 (IL-2) following local BM-treatment may confer survival benefits [7]. These observations suggest that effective medical treatment may account for the outcome of BM-patients rather than the diagnosis of BMs per se.

Therapeutic options for mRCC have tremendously improved in the last 3 years [8-11]. When compared to interferon-alpha, first-line treatment with the tyrosine kinase inhibitor (TKI) sunitinib was shown to significantly improve objective remission rates (39% versus 12%), progression free survival (11 months versus 5 months) and overall survival (26.4 months versus 21.8 months) [12]. Similarly, first-line treatment with the monoclonal antibody bevacizumab in combination with interferon-alpha was shown to provide a statistically significant benefit in overall response rate (ORR) (31% versus 13%) and PFS (10.2 months versus 5.4 months) [11] when compared to IFN-alpha alone. In poor risk patients, treatment with the mammalian target of rapamycin (mTOR)-inhibitor temsirolimus was associated with a statistically significant benefit in OS when compared to patients treated with IFN-alpha (10.9 months versus 7.3 months) [10]. In second-line, the tyrosine kinase inhibitor sorafenib was shown to double PFS (5.5 versus 2.8 months, HR: 0.44, 95%CI 0.35-0.55, p < 0.01) when compared to placebo in patients who had progressed on cytokine treatment [9]. Finally, the oral mTOR inhibitor everolimus was shown to reduce the risk for progression in patients who had failed Vascular endothelial growth factor receptor (VEGFR)-based TKI first-line treatment (HR 0.30, 95%CI: 0.22-0.40) [13]. Patients with BM were excluded from these pivotal trials, however, subsequently initiated smaller studies also investigated the outcome of BM-patients and several authors have shown that sunitinib and sorafenib can be given safely, i.e. without haemorrhage in patients with BMs [6,14-18].

With the advent of these novel agents, extracerebral disease control is enabled in the majority of patients. As patients with BM are endangered by distant metastases rather than brain metastases, we hypothesized that response rates, progression-free survival and overall survival should be similar in patients with and without BM. The aim of this retrospective analysis was to compare the outcome of patients with and without BM since the start of the era of targeted agents and to investigate whether progression of brain metastases is the most limiting factor for overall survival.

## Methods

All patient data were collected at the Department of Medicine I and Cancer Center, Clinical Division of Oncology at the Medical University of Vienna. This retrospective analysis was performed in accordance with the ethical regulations of the Medical University of Vienna.

### Patients

Data from 114 consecutive mRCC patients who had access to at least one type of targeted agent during their course of the disease were recorded and analyzed for presence of BM at the start of the first targeted agent, response to treatment, progression free survival, overall survival and cause of death. The first novel agent provided and reimbursed was bevacizumab, which was available for off label use in October 2005. Sunitinib, sorafenib, temsirolimus were available later, i.e. in February 2006, June 2006 and March 2007, respectively. The choice for a specific medical treatment was first based on the availability of these agents and later on the results of the pivotal trials. Sunitinib and sorafenib were prescribed at a daily dose of 50 mg (on a 4 weeks on, 2 weeks off schedule) and 800 mg/d (continuously), respectively. Temsirolimus was prescribed at a weekly dose of 25 mg and bevacizumab every 2 weeks at 10 mg/kg body weight. Staging investigations were performed at baseline and every 3 months and earlier if clinically required. Response to treatment was evaluated according to the Response Criteria in Solid Tumors (RECIST) [19]. The diagnosis of BMs was made by computed tomography (CT) scan or magnet resonance tomography (MRI).

### Statistical methods

OS was calculated from the start of the first targeted therapy until death. Survival was calculated using the Kaplan-Meier method and groups (no brain metastases versus brain metastases at start of first targeted agent) were compared with the log-rank test. Patient characteristics were compared between the different groups by using the Chi-Square test and the Fisher's Exact test. Multivariate analyses were performed with Cox-Regression. A two-sided p-value lower or equal 0.05 represents significance in all tests.

## Results

Patient characteristics are outlined in table 1. Between October 2005 and February 2009, 114 consecutive patients with a median age of 65.5 years (range 45-88 years) had access to at least one targeted agent during their course of the disease. The majority of the patients were in good performance status (ECOG 0: 72.8%) and were intermediate risk (58.7%) according to the Memorial Sloan-Kettering Cancer Center (MSKCC) criteria [20]. The most common site of metastases was the lung (69.3%) followed by bone (36%) and lymph nodes (33.3%). 47.4% of the patients had 2 metastatic sites. All but 6 patients had undergone nephrectomy. Sixty-five out of 114 patients (57%) had prior therapies for metastatic disease consisting of cytokines and/or chemotherapy, another forty-nine patients (43%) were treatment naïve. The first targeted agents were mostly sunitinib (63.2%) and sorafenib (30.7%). Another 4 and 3 patients received bevacizumab and temsirolimus as the first targeted agent. Twelve out of 114 patients (10.5%) had - in addition to extracerebral metastases- BM at baseline and underwent local treatment before targeted agents were offered.

**Table 1:** Baseline characteristics of renal cell carcinoma patients (n = 114)

Characteristics	N	%
**Sex**		
male	69	60.5
female	45	39.5

**Age**		
median (range)	65.5 (45-88)	47.4
< 65	54	52.6
> 65	60	

**ECOG**		
0	83	72.8
1	28	24.6
2	3	2.6

**Nephrectomy**		
Yes	108	94.7
no	6	5.3
		

**Time from primary to metastasis**		
< 1 year	56	49.1
> 1 year	58	50.9

**Histology**		
clear cell	104	91.2
non clear cell	10	8.8

**MSKCC**		
favourable	45	39.5
intermediate	67	58.7
poor	2	1.8
		

**Number of metastasis**		
1	32	28.1
2	54	47.4
> 3	28	24.5

**Location of metastasis**		
Lung	79	69.3
Liver	22	19.3
Bone	41	36
Lymph nodes (LN)	38	33.3
CNS	12	10.5
other	41	36

**Systemic therapy before first Targeted Agent (TA)**		
Cytokines	54	47.4
other	11	9.6

**First targeted agent**		
Bevacizumab	4	3.5
Sunitinib	72	63.2
Sorafenib	35	30.7
Temsirolimus	3	2.6

### Specific characteristics of BM patients at diagnosis of brain metastases

Specific characteristics of BM patients are shown in Table 2. The median age of BM patients was 66 years. All BM-patients had extracerebral metastases (clear cell n= 2, non clear-cell n = 10), 41.7% in 2 or more other sites. The most common sites were the lung (67%) and lymph nodes (25%). Six patients (50%) had 2 or more cerebral lesions. Brain metastases were mostly located in the cerebellum (33.3%) or the frontal lobe (33.3%). In 75% of BM patients, at least one lesion was larger than 2 cm. The majority of BM-patients (83%) were symptomatic at diagnosis of BM, the most common symptoms being headache and ataxia. All patients diagnosed with BMs underwent local treatment (surgery only: n = 2; stereotactic radiation only: n = 2; whole brain radiation only n = 3, combinations: n = 5).

**Table 2:** Specific characteristics of BM patients (n = 12)

Age (median, range)	66 (45-88)	
	**n**	**%**

**Number of extracerebral metastatic sites at occurrence of BM**		
- 1	7	58.3
- 2	2	16.7
- ≥ 3		

**Location of extracerebral metastases**		
Lung	8	66.7
Liver	2	16.7
Bone	2	16.7
LN	3	25
other	5	41.7

**Time from primary tumor to occurrence of BM**		
< 2 years	9	75
> 2 years	3	25

**Number of brain lesions**		
1	6	50
2	4	33.3
> 2	2	16.7

**Location of BM metastases**		
- frontal	4	33.3
- parietal	2	16.7
- cerebellar	4	33.3
- temporal	1	8.3
- dissiminated	1	8.3

**Lesion size**		
1cm	1	8.3
1-2cm	2	16.7
> 2cm	9	75

**CNS symptoms**		
Yes	10	83.3
No	2	16.7
- psychiatric	1	8.3
- vertigo	2	16.7
- headache	5	41.7
- ataxia	3	25
- hemiplegia	1	8.3

**Local treatment for BM**		
- surgery	2	16.7
- surgery+ SR	1	8.3
- surgery+WBRT	2	16.7
- SR	2	16.7
- SR+WBRT	2	16.7
- WBRT	3	25
		

**First targeted agent after local control of BM**		
- Bevacizumab	2	16.7
- Sunitinib	7	58.3
- Sorafenib	2	16.7
- Temsirolimus	1	8.3

### Response to Treatment, Progression Free Survival and Overall Survival

**Figure 1:**
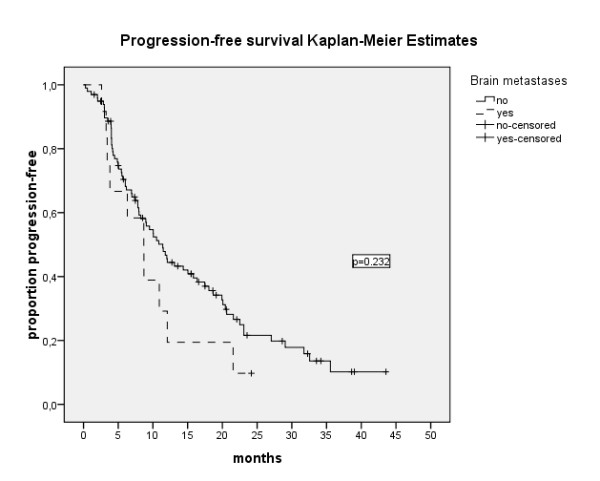
**Progression free survival of 114 patients with and without BM**. Median PFS: 10.9 months (95%Ci: 8.3-13.5), 11.4 months (95% CI: 8.7-14.1) and 8.7 (95% CI: 5.1-12.3) for the entire population, non-BM-patients and BM-patients, respectively (p = 0.232).

**Figure 2:**
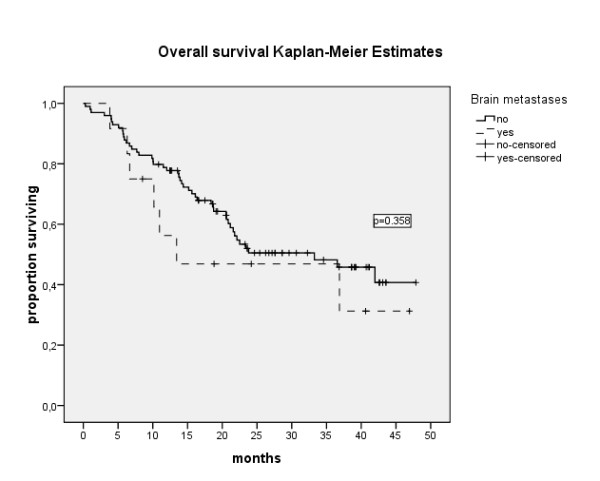
**Overall survival of 114 patients with and without BM**. Median OS: 33.3 months (95%CI: 19.3-47.2), 33.3 months (95%CI: 18.6-47.9) and 13.4 months (95%CI: 1-43.9) for the entire population, for non-BM and BM patients, respectively (p = 0.358).

As outlined in table 3, 33 out of 109 evaluable patients (30.3%) achieved objective remission under their first targeted agent (complete remission (CR): n = 6, partial remission (PR): n = 27). Another 61 patients (56%) achieved stable disease (SD) for more than 3 months. Three out of 12 BM-patients (25%) achieved objective remission (CR: n = 1, PR: n = 2). Another seven patients (58.3%) had SD for at least 3 months. The PFS was 10.9 months (range 8.3-13.5), 11.4 months (8.7-14.1) and 8.7 (5.1-12.3) for the entire population, non-BM-patients and BM-patients, respectively (p = 0.232) (Figure 1). The median overall survival was 33.3 months (95%CI: 19.3-47.2), 33.3 months (95%CI: 18.6-47.9) and 13.4 months (95%CI: 1-43.9) for the entire population, for non-BM and BM patients, respectively (p = 0.358) (Figure 2).

**Table 3:** Outcome of renal cell carcinoma patients with and without cerebral metastasis

	All patients n = 109 n	%	Patients without BM n = 97 n	%	Patients with BM n = 12 n	%
**Best response to TA**						
- CR	6	5.5	5	5.2	1	8.3
- PR	27	24.8	25	25.8	2	16.7
- SD	61	56	54	55.7	7	58.3
- PD	15	13.8	13	13,4	2	16.7

	**All patients n = 114** **median (months)**	**95%CI**	**Patients without BM** **n= 102 median (months)**	**95% CI**	**Patients with BM n = 12 median (months)**	**95% CI**

**Progression free survival **(start first TA- progression)	10.9	8.3-13.5	11.4	8.7-14.1	8.7	5.1-12.3

**Overall survival **(start first TA- death)	33.3	19.3-47.2	33.3	18.6-47.9	13.4	1-43.9

BM = brain metastases

TA = targeted agent

CR = complete response

PR = partial response

SD = stable disease

PD = progressive disease

The individual course of disease of BM-patients is outlined in table 4. Three out of 12 BM-patients had a central nervous system (CNS)-relapse and underwent additional local treatment. None of the BM-patients has been diagnosed with or died from BM-specific disease progression since local treatment. ECOG Performance status and the time from primary tumor to metastases were independent risk factors for short survival while no patients died from cerebral disease progression (ECOG Performance status 0: 42 months 95%CI: 19.33-47.2, ECOG >1: 16.4 months, 95%CI: 10.3-22.5, Hazard ratio (HR 2.74, p = 0.001), TDM <1 year: 20.6 months, 95%CI: 14.1-27.1, and TDM >1 year: 42 months, 95%CI 32.8-51.2, HR: 0.552, p = 0.034).

**Table 4:** Individual course of disease of BM patients

Patient	MSKCC risk group	ECOG performance status at diagnosis of BM	Extracerebral metastatic site	TX prior first TA	Time from primary metastasis to BM (months)	BM-TX	CNS recurrence during/after TA	First TA	Best Response	PFS	Following TX lines	OS	Progressing metastatic site leading to death
1	fav	0	Lung	Cytokines	0	surg	no	SU	CR	24.2	Ongoing	24.2	non progressing and alive

2	interm	0	Lung	Cytokines, Capecitabin	45	surg	no	Beva	SD	8.7	SU Soraf Tems Tems+Beva	46.9	non progressing and alive

3	interm	1	lung, LN	no	0	surg, WBRT	no	SU	SD	8.5	ongoing	8.5	non progressing and alive

4	interm	1	lung, other	Cytokines	72	SR	no	Beva	SD	21.6	SU, Tems	36.9	lung, liver

5	fav	0	liver, bone, other	Cytokines, Navelbine	27	WBRT, SR	yes (progression in lesion size)	Soraf	PD	2.6	SU	13.42	lung.

6	interm	1	other	no	0	SR, WBRT	yes (progression in lesion size)	SU	PD	3.4	Tems, Beva +Interferon	10.16	Liver

7	interm	0	Ln	no	0	WBRT	no	SU	PR	3.26	Soraf	18.82	non progressing and alive

8	interm	1	lung	no	0	Surg, SR	no	SU	SD	12.1	SU	40.6	non progressing and alive

9	interm	1	lung, liver, other	no	0	SR	no	Tems	SD	6.3	no	6.3	liver, lung

10	interm	0	lung, ln	no	8	WBRT	no	Soraf	PR	10.9	no	10.9	Lung

11	interm	0	lung, other	Cytokines, Navelbine	14	Surg,WBRT	yes (relapse of resected lesion and new lesions)	SU	SD	3.8	no	3.8	cranial vertebral fractures

12	poor	2	bone	no	0	WBRT	No	SU	SD	6.7	no	6.7	Lung

MSKCC = Memorial Sloan-Kettering Center Center

fav = favourable risk

interm = intermediate risk

poor = poor risk

ECOG = Eastern Cooperative Oncology Group

TX = treatment

TA = targeted agent

BM = brain metastasis

SR = stereotactic radiation

WBRT = whole brain radiation therapy

Surg = surgery

SU = Sunitinib

Soraf = Sorafenib

Tems = Temsirolimus

Beva = Bevacizumab

CR = complete remission

PR = partial remission

SD = stable disease

PD = progressive disease

PFS = progression free survival

OS = overall survival

### CNS-related PFS and BM recurrence

The median CNS-related PFS (start of local BM-treatment to BM recurrence) is 12.37 months (95% CI: 1-49.76). Three out of 12 patients (25%) had a BM recurrence. Two patients had a progression in lesion size after local treatment and one patient had a relapse of the resected metastasis and new CNS lesions.

### Toxicity and treatment-related side effects of BM and non-BM patients

The most common grade 3 and 4 toxicities in BM-patients were diarrhoea (22.2%), nausea (11.1%) and hypertension (11.1%). In non-BM patients hypertension (19.6%), hand/foot-syndrome (17.6%) and diarrhoea (12.8%) were the most common grade 3 and 4 toxicities.

## Discussion

In the era of cytokine treatment, extracerebral metastases rather than brain metastases were shown to determine the course of disease in patients with advanced RCC and brain lesions. Most patients with BM either did not benefit from cytokine treatment or were considered unsuitable for cytokines [21]. The advent of targeted agents for RCC has enabled significantly better control of systemic disease [8-11, 13]. Thus we hypothesized that patients with and without BM may have similar benefits from modern agents. We found that targeted agents led to quite similar response rates in patients with and without BM. However, the median progression free survival and overall survival were strikingly lower in patients with BM when compared to patients without (PFS: 8.7 versus 11.4 months, p = 0.232, OS: 13.4 months versus 33.3 months, p = 0.358). A limitation of our report is the variety of local and systemic therapies offered (i.e. stereotactic radiosurgery or WBRT, sunitinib, sorafenib, bevacizumab and temsirolimus). Moreover, differences in PFS and OS did not reach statistical significance due to the small number of patients. However, our findings are consistent with other reports. In the sunitinib extended access program, the median progression free survival and overall survival for the entire population were 10.9 and 18.4 months, respectively, whereas the subgroup of patients with BM was shown to achieve a median PFS and OS of 5.6 and 9.2 months, only [6].

Although (radio-) surgery was shown to provide excellent local control in up to 96% of the patients [1,22], it would be expected that reduced overall survival of BM-patients is related to the tenuous site of metastases. This is supported by the report of Sperduto and colleagues who found that the number of cerebral lesions is an independent prognostic factor in patients with RCC [23]. Brain metastases may dramatically endanger the patient by leading to local edema, increased intracranial pressure and fatal bleeding. However, in our series, brain metastases per se were not responsible for shorter survival of these patients. All patients with BMs were neurologically unsuspicious in the last 24 hours before death and died obviously from disease progression of extracerebral metastatic sites, leading most commonly to respiratory or hepatic failure. Among those who are alive, none is endangered by intracerebral disease progression. Hence, the presence of BM was not a risk factor for shorter overall survival (p = 0.358). In the multivariate analysis, only ECOG Performance status (ECOG 0: 42 months 95%CI: 19.33-47.2, ECOG >1:16.4 months, 95%CI: 10.3.22.5, HR: 2.74, p = 0.001) and the time from primary tumor to development of metastases (< 1 year: 20.6 months, 95%CI 14.1-27.1, and > 1 year: 42 months, 95%CI 32.8-51.2, HR: 0.552, p = 0.034) were independent risk factors for short survival. Our results are consistent with the findings of Hara et al. who reported that a poor performance status accounts for shorter survival of BM-patients rather than the presence of brain metastases per se [22]. Consequently the authors suggested that effective therapeutic strategies for systemic disease may prolong survival in patients with locally treated brain metastases.

Although all patients of the present analysis had access to at least one type of effective RCC treatment leading to stable disease or objective remission in the majority and while no patient died from BM-related progression, the outcome is still different between BM and non-BM-patients. Several reasons may account for these differences. First, brain metastases were shown to occur late in the progression of mRCC [24]. Consequently, the overall survival of these patients may often reflect only the length of the very last life span within the course of metastatic disease. Second, the occurrence of brain metastases may represent an epiphenomenon of an altered, meanwhile highly aggressive behaviour of the tumor. Hence, it could be speculated that patients with BM require far more aggressive treatment strategies than patients without and that alternative therapeutic targets may become more relevant. In this context, the signal transducer and activator of transcription 3 (Stat3) might be an interesting target. Activation of Stat3 was shown to be increased in brain metastases [25]. Sunitinib inhibits Stat3 in both tumor cells and tumor myeloid cells [26], thereby leading to tumor cell apoptosis and reduced expression of angiogenic genes and immunosuppressive cells. However, persistent Stat3 activation has been shown to rescue tumor cells from sunitinib-induced cell death [26] and to promote cell proliferation by regulating genes encoding antiapoptotic and proliferation-associated proteins [27]. Patients with brain metastases and activated Stat3 may require a treatment strategy that inhibits Stat3 in various ways. A combination of sunitinib with an anti-interleukin-6 antibody has been suggested, since Stat3-activation is mediated by the interleukin-6 receptor [26]. Another promising target whose expression correlates with advanced stage of disease is the chemokine receptor CXCR4, a key receptor in the crosstalk between tumor cells and their environment [28]. In RCC, the loss of function of the Von Hippel Lindau (*VHL*) tumor supressor gene mediates up-regulation of CXCR4 [29] which then promotes tumor spread and progression [28]. The activity of CXCR4-directed agents has already been shown in animal tumor models [30] and might be of particular interest in rapidly progressing tumors.

## Conclusions

Despite both excellent neuro(radio-)surgical strategies and advances in the treatment of systemic mRCC, the course of disease of patients with brain metastases remains an enormous challenge. These patients are endangered by rapidly progressing extracerebral metastases rather than progressing brain metastases. Thus, different i.e. more complex therapeutic concepts are urgently required for this patient population and baseline and repeated CT-scans of the brain should be provided in all patients in order to enable highly potent local treatment options.

### Declaration of competing interests

Manuela Schmidinger has acted as an adviser to Pfizer, Bayer-Schering, Wyeth, Novartis, Roche and GSK, has received research grants from Pfizer and Wyeth, travel grants from Bayer-Schering and Wyeth and lecture fees from Pfizer, Bayer-Schering, Novartis, Roche and Wyeth. Christoph Zielinski has acted as an adviser to Pfizer and Roche, has received lecture fees from Pfizer, Merck, Lilly and Roche and research grants from Pfizer and Wyeth. Gero Kramer has received research grants from Sanofi Aventis, Bayer-Schering and Takeda, lecture fees from Sanofi Aventis, Astra Zeneca and Astellas, acted as an advisor to Sanofi Aventis and received travel grants from Pfizer, Boehringer-Ingelheim, Sanofi Aventis and Bayer-Schering. Ursula Vogl has received travel grants from Pfizer, Wyeth Roche and Bayer-Schering. Wolfgang Lamm has received travel grants from Bayer-Schering. Oskar Pichelmayer has received research grants from Roche and travel grants from Bayer-Schering. Marija Bojic, Andrea Haitel, Josa Frischer, Kaan Harmankaya and Klaus Kitz have no conflicts of interest to declare.

## Authors' contributions

UMV originally conceived of the study design, recruited the patients, collected and reviewed the clinical data, analyzed and interpreted the results, wrote the manuscript, participated in table and figure conception and oversight the entire project. MB participated in patient recruitment, collection and review of clinical data, clinical data analysis, clinical figure design, project coordination and biostatistical analysis. WP participated in patient recruitment, collection and review of clinical data and project coordination. WL participated in patient recruitment, collection and review of clinical data and project coordination. JMF participated in clinical data analysis, clinical table and figure design and biostatistical analysis. AH participated in patient recruitment, collection and review of clinical data, clinical data analysis and interpretation and project coordination. GK participated in clinical study design, collection and review of clinical data, clinical data analysis and interpretation and project coordination. KH participated in patient recruitment, collection and review of clinical data and clinical data interpretation. MS and CCZ participated in patient recruitment, review of clinical data, clinical data interpretation, manuscript writing and project coordination.

All authors have and approved the final manuscript.

## Role of the funding source

The study was sponsored by the Medical University of Vienna, who had no involvement in the design, conduct, data collection and data analysis. The corresponding author had access to all data and takes responsibility for the accuracy and completeness of the data reported. The corresponding author had final responsibility for the decision to submit for publication.

## Pre-publication history

The pre-publication history for this paper can be accessed here:

http://www.biomedcentral.com/1471-2407/10/480/prepub
